# Comparative study of the usefulness of a novel insulin therapy in Japanese patients with Type 2 diabetes for concomitant use of an oral antidiabetic agent with twice-daily dosing either of insulin aspart, biphasic insulin aspart-30, or insulin detemir: Two times Insulin injection Combined with oral therapy Efficacy Study (TWICE study)

**DOI:** 10.20407/fmj.2019-023

**Published:** 2020-07-14

**Authors:** Mitsuyasu Itoh, Atsushi Suzuki, Taiya Katoh, Yoshikuni Sawai, Shogo Asano, Shigeo Imamura, Yoshinari Hayashi, Shinobu Goto, Nobuo Takahashi, Tetsuya Kawabe

**Affiliations:** 1 Department of Endocrinology and Metabolism, Fujita Health University, School of Medicine, Toyoake, Aichi, Japan; 2 Department of Internal Medicine, Toyota Kosei Hospital, JA Aichi Koseiren, Toyota, Aichi, Japan; 3 Department of Internal Medicine, Toyokawa City Hospital, Toyokawa, Aichi, Japan; 4 Endocrinology and Metabolism, Kariya Toyota General Hospital, Kariya, Aichi, Japan; 5 Department of Internal Medicine, Division of Metabolism and Endocrinology, Nagoya Memorial Hospital, Nagoya, Aichi, Japan; 6 Takahashi Family Clinic, Nagoya, Aichi, Japan; 7 Kawabe Clinic, Division of Internal Medicine and Dermatology, Okazaki, Aichi, Japan

**Keywords:** Type 2 diabetes, Twice daily, Long-acting, Premixed, Insulin

## Abstract

**Objectives:**

It is common to treat type 2 diabetes by regular injections of insulin. We compared the efficacy and safety of twice-daily administration of short-acting, premixed, and long-acting insulins.

**Methods:**

This was a multi-center, randomized, open-label, 52-week study. Patients were randomized to administer twice daily short-acting analog insulin (Aspart) plus a sulfonylurea (SU), premixed 70/30 analog insulin (Mix), or long-acting insulin (Detemir) plus a glinide derivative.

**Results:**

Twelve (mean baseline HbA1c 9.86±1.71%), eight (9.24±1.14%), and eight (11.26±1.81%) patients were treated with Aspart, Mix, or Detemir, respectively, for 52 weeks. After 12 weeks, the reductions in HbA1c were similar in the groups. A further significant reduction in HbA1c occurred between weeks 12 and 52 in the Detemir, but not the Aspart or Mix groups. After 52 weeks, the target of an HbA1c <7.4% was achieved in 16.7% of the Aspart group, 37.5% of the Mix group, and 12.5% of the Detemir group (no significant differences among the three groups by χ^2^ analysis). The mean changes from baseline in blood glucose concentration measured after breakfast, and before and after dinner, were also similar in each group.

**Conclusions:**

Early and meaningful reductions in HbA1c were achieved by twice-daily administration of a premix, aspart plus an SU, and detemir plus a glinide, without severe hypoglycemia or an increase in body mass. However, the target HbA1c was achieved in relatively few participants, perhaps due to an insufficient dose of insulin or the small study size.

## Introduction

It is important for diabetic patients to maintain good glycemic control, to prevent diabetic complications developing.^1–3^ To achieve target blood glucose concentrations, insulin therapy for type 2 diabetes (T2DM) may be necessary if the administration of oral antihyperglycemic agents (AHAs) alone is insufficient, as suggested by the European Association for the Study of Diabetes (EASD)/American Diabetes Association (ADA) statement.^4^ It has been reported that basal-bolus insulin regimens resemble the physiological pattern of insulin secretion, and that their use results in a smoother 24-h glucose profile, compared with conventional insulin regimens.^5^ However, these regimens may induce hyperinsulinemia, which can accelerate atherosclerosis and increase the risk of hypoglycemia.

A further complication of such regimens is that three or more daily injections may be required. It is still popular in Japan to treat T2DM by twice-daily injections of insulin, before breakfast and dinner; the administration of an injection before lunch can be difficult, because of work commitments. Furthermore, the Diabetes Attitudes, Wishes, and Needs (DAWN) Japan study revealed that many factors make it difficult for patients to treat T2DM using multiple injections of insulin, including personal as well as social barriers.^6^

We aimed to determine the efficacy and safety of twice-daily injections of various analogs of insulin, in addition to the oral administration of anti-diabetic drugs, by comparing short-acting Aspart^®^ with long-acting Detemir^®^ and the premixed 30 Mix^®^ analog (30/70 Aspart^®^/Detemir^®^). The insulin analogs were administered twice daily, to avoid a higher requirement in the morning, the need for a big meal in the evening, and hypoglycemia in the afternoon due to physical activity, for 12 weeks. The efficacy of each regimen was evaluated on the basis of whether additional treatment was required after 4 weeks. The purpose and the methods used were specified in the design of the TWo times Insulin injection Combined with oral therapy Efficacy (TWICE) study. However, the full study was not completed, because the required number of patients were not recruited during the enrollment period. Therefore, the present paper presents the results of a smaller study conducted during the originally specified trial period.

## Methods

### Study design

We conducted a multi-center, randomized, open-label, 52-week study. The objective was to compare the efficacy of twice-daily insulin therapy (insulin aspart, biphasic insulin aspart-30, or insulin detemir) in combination with an oral antidiabetic agent in eligible Japanese T2DM patients <75 years of age who were being treated using diet, exercise, and an oral AHA. The additional inclusion criteria were as follows: T2DM of >1 year, HbA1c concentration (National Glycohemoglobin Standardization Program) of 7.5%–10.5%, stable AHA treatment for ≥12 weeks, fasting blood glucose concentration (FBG) >140 mg/dl, and body mass index (BMI) <35 kg/m^2^. Patients who were being treated with insulin at an outpatient clinic, whose BMI was >35 kg/m^2^, or who had severe proteinuria or neuropathy were excluded from the study. Patients received diet and exercise counseling throughout the study. Thirty-one patients were enrolled after written informed consent was obtained.

The primary outcome was an HbA1c <7.4%. The key secondary outcomes were the safety and dose of insulin administered, the change in casual BG, the change in and mean value of BG before breakfast, BG 2 h after breakfast, BG before dinner, BG after dinner, efficacy evaluated by the physician in charge, and change in body mass. The target sample size was 100.

Adverse events were recorded by the physician in charge at every patient visit and when reported by the patient. The pre-specified adverse events were severe hypoglycemia, heart failure, liver dysfunction, and related laboratory data.

Eligible patients were randomized using a numbered container method to administer a short-acting analog insulin (Aspart) plus a sulfonylurea (SU), a premixed 70/30 analog insulin (Mix), or a long-acting insulin (Detemir) plus a glinide derivative insulin-releasing agent, administered twice-daily. Prescribed pioglitazone or metformin were continued throughout the study, but other AHAs were discontinued. Reductions in the doses of oral AHA or insulin were made as required by the physician in charge.

After a period of administration of a fixed dose, rescue therapy was initiated using short-acting insulin before lunch. The regimen and dose were determined by the physician in charge; rescue therapy was administered if FBG was >200 mg/dl at any time between randomization and week 52. Of the 31 patients recruited, three dropped out of the study.

### Patients

Patients with T2DM were eligible to participate if they had a baseline HbA1c of 7.8%–10.4%, and they were 20–75 years old at the screening visit.

The study was performed in accordance with Good Clinical Practice standards and the ethical principles derived from the Declaration of Helsinki. The study protocol was reviewed and approved by the ethics review committee of Fujita Health University (10-210) and the other appropriate committees and authorities. All the patients provided their written informed consent to participate. This clinical trial was registered as UMIN000003537.^7^

### Efficacy assessments

The primary efficacy end-points were the change in HbA1c from baseline and the percentage of participants with an HbA1c <7.4% after 12, 24, and 52 weeks. Other efficacy assessments included BG before and after breakfast and dinner, and before bedtime, and serum LDL- and HDL-cholesterol, and triglyceride concentrations.

### Safety and tolerability assessments

The safety measurements made included evaluations of adverse events and physical examination by the physician in charge at every patient visit. The pre-specified adverse events were severe and non-severe hypoglycemia, heart failure, liver dysfunction, renal dysfunction, and related laboratory data. The laboratory safety studies included blood chemistry, hematology, and urinalysis.

### Blood collection and assays

Participants were instructed to fast overnight for 8 h, prior to collection of blood for laboratory assessment. Blood was collected at baseline and after 12, 24, and 52 weeks of treatment, for efficacy and safety measurements.

### Statistical analysis

The primary study end-points were the change in HbA1c from baseline, the prevalence of hypoglycemic events, and the overall safety and tolerability. The secondary end-points were the changes in body mass and FBG from baseline. The primary time point was week 52. The percentage of participants achieving an HbA1c <7.4% as the primary outcome were compared among the three groups by χ^2^ analysis after 12, 24, and 52 weeks.

The primary efficacy analysis population was the per-protocol population, which comprised all the randomized patients who had the appropriate end-points assessed at baseline and after 12, 24, and 52 weeks of treatment, and did not deviate significantly from the protocol. Statistical analysis was performed by one-way ANOVA, followed by Bonferroni’s adjustment, Dunnett analysis, or Wilcoxon/Kruskal-Wallis analysis, as applicable, using JMP^®^10 (SAS Institute Inc.). A p value <0.05 was considered significant.

## Results

### Patient characteristics and disposition

The patient characteristics are shown in Table 1. Overall, 31 patients were recruited and randomized. The trial commenced on May 1, 2010, and patients remained on the intervention therapy for 52 weeks. The final follow-up date was December 1, 2011. Thirteen patients were treated with Aspart plus an SU (Aspart group), nine were treated with Mix (Mix group), and nine were treated with Detemir plus a glinide (Detemir group). The mean baseline HbA1c was 9.86±1.71 for the Aspart group, 9.24±1.14% for the Mix group, and 11.26±1.81% for the Detemir group. Twelve patients treated with Aspart, nine patients treated with Mix, and eight patients treated with Detemir completed the full 52 weeks of the study. One patient treated with Mix achieved good glycemic control and discontinued his insulin after 15 weeks (Table 1).

### Efficacy

The reduction achieved in HbA1c in each group is shown in Table 2. After 12 weeks, the mean HbA1c in the Mix group was the lowest of the three groups, and was significantly lower than that of the Detemir group. However, the reductions from baseline (1.52±1.69% for Aspart, 1.73±1.11% for Mix, and 1.98±1.58% for Detemir groups) in HbA1c were not statistically significant in each treatment group. At week 24, similar reductions from baseline were also observed (1.58±1.74% for Aspart, 1.59±1.07% for Mix, and 2.21±2.30% for Detemir groups). A further significant reduction in HbA1c was measured in the Detemir group between weeks 12 and 52, but no further reduction was observed in either the Aspart or Mix groups. The reductions in HbA1c at 52 weeks were 1.43±2.07% in the Aspart group 1.46±1.42% in the Mix group, and 2.94±1.88% in the Detemir group; there were no significant differences in this reduction among the three groups (Table 2).

The primary outcome was the percentage of participants achieving an HbA1c <7.4%. The percentages obtained were 16.7% for Aspart, 25.0% for Detemir, and 55.6% for Mix after 12 weeks. After 52 weeks, the percentages were 16.7% for Aspart, 37.5% for Mix, and 12.5% for Detemir. Thus, the Mix group contained the highest percentage of participants achieving the target, but there was no significant differences among the three groups by χ^2^ analysis (*p*=0.057). The percentage declined to 33.3% and 37.5% at weeks 24 and 52, respectively, in the Mix group, but these were still higher than those achieved in the Aspart and Detemir groups (Table 3).

**Figure 1:**
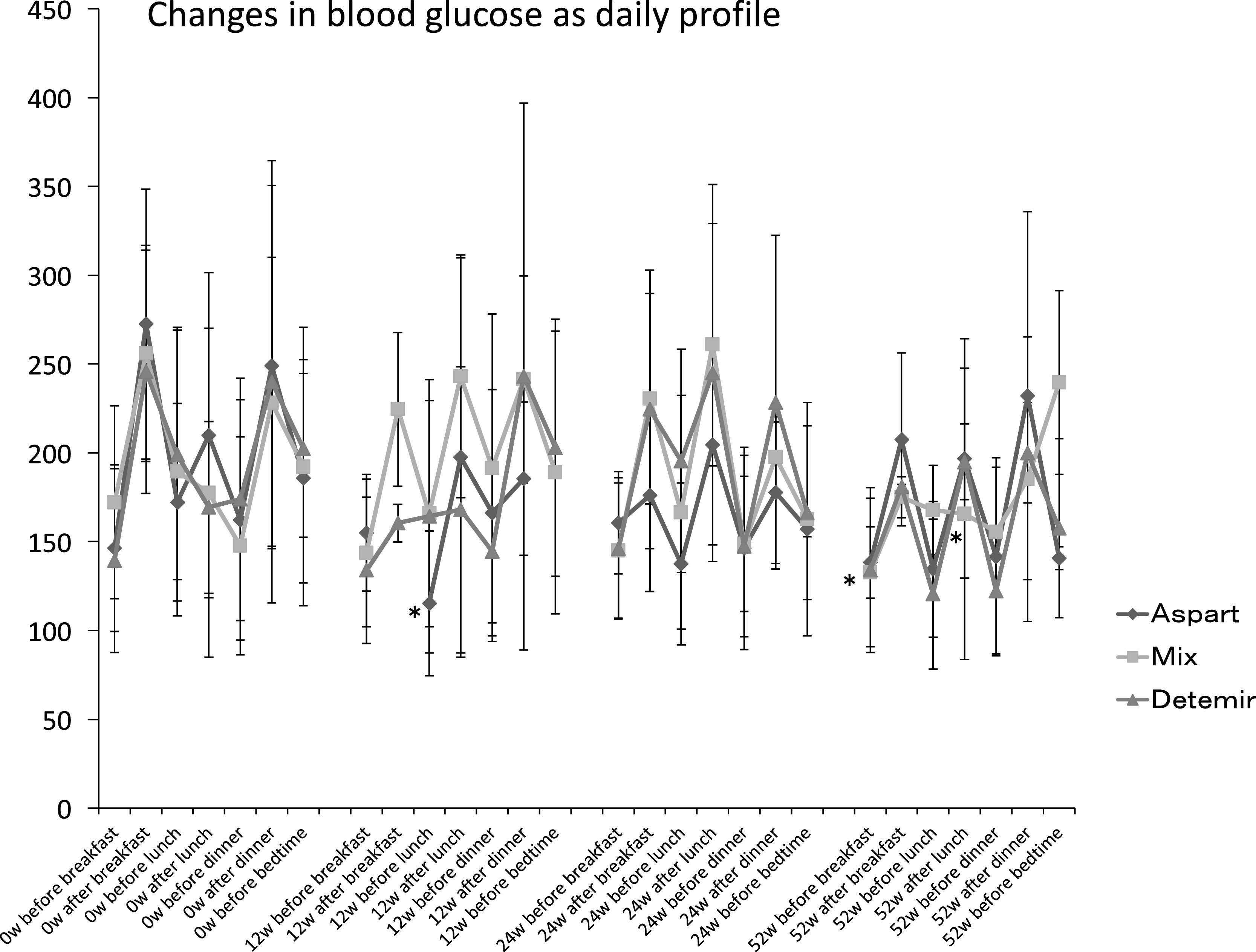
Daily blood glucose profile Mean±standard deviation (SD), * *p*<0.05 *vs.* 0 w. W: week, Mix: twice-daily dosing of biphasic insulin aspart-30.

A significant reduction in BG before lunch was measured in the Aspart group at week 12, and in BG after lunch in the Mix group at week 52 (Figure 1). A reduction from baseline in FBG was measured at week 52 in the Mix group, but there were no reductions in the Aspart or Detemir groups at weeks 12, 24, or 52. The BG profiles, showing the mean change from baseline in BG after breakfast, and before and after dinner, were similar for each group.

There was no statistically significant change in body mass in any of the treatment groups (Table 4). The daily dose of insulin in the Mix group was higher than that of the Aspart group at the start of the study (0 weeks), because the Mix contained 30% aspart and 70% intermediate-acting insulin. The daily dose of insulin was significantly higher at week 24 in the Aspart group and at weeks 12 and 52 in the Detemir group, but no significant increases were necessary in the Mix group. There were no significant differences in dose among the three groups at week 52 (Table 5). Throughout the 52-week treatment period, four of thirteen (30.8%) patients in the Aspart group and two of nine (22.2%) in the Mix group had started on glycemic rescue therapy with insulin before lunch, whereas no additional insulin was administered by the Detemir group.

In the Detemir group, there were decreases from baseline in serum LDL-cholesterol and triglyceride concentrations after 12 and 52 weeks of the study (Table 6).

According to the final judgment of the physician in charge, the efficacy rates were 66.7% for Mix, 50% for Aspart, and 16.7% for Detemir.

### Safety

The insulins and glinides were generally well-tolerated over the 52 weeks of the study. The overall incidences of adverse effects were similar among the three groups. There were no severe cases of hypoglycemia, which was defined as the necessity for assistance by medical staff or hospitalization. There were no adverse effects detected using liver enzyme assays, serum electrolyte measurement, or hematology in the three groups throughout the study period (Table 6). One participant in the Mix group was reported to have developed heart failure, but the physician in charge decided that this was related to the patient’ pre-existing coronary heart disease, rather than the study protocol, and therefore they continued their participation to the end of study.

## Discussion

This study has demonstrated significant reductions in HbA1c in patients with T2DM who were treated with one of twice-daily pre-mixed insulin, rapid-acting insulin plus a SU, or detemir plus a glinide. These reductions were rapid, and maintained in participants using the pre-mixed or rapid-acting insulin, whereas they continued after 24 weeks in participants using detemir. The percentage of participants achieving an HbA1c <7.4% was higher in participants in the Mix group than the Aspart or Detemir groups, although the difference did not reach statistical significance. The explanation for the relatively poor percentages may be that insufficient doses of insulin were administered to overcome hyperglycemia, because adverse effects, such as severe hypoglycemia or a significant increase in body mass, were not observed. To increase the efficacy of the therapeutic regimens, higher doses of insulin may be required, although the risks of hypoglycemia and body mass gain would also increase. At the initiation of the study, the daily dose given by the Aspart group was lower than that of the Mix group, because of the rapid action of aspart compared to the premix (30% rapid and 70% intermediate-acting) and detemir (no rapid-acting) insulin. However, after 12 weeks, the daily doses of insulin were similar, suggesting that the dose of insulin at the start was not enough to overcome post-prandial hyperglycemia. Further study is required to identify the optimal dose of insulin to achieve a sufficient reduction in HbA1c, without adverse effects. Overall, the efficacy of the regimens, as judged by the physician in charge, was 66.7% in the Mix group, 50% in the Aspart group, and 16.7% in the Detemir group. Severe hypoglycemia was not observed, and mean body mass did not change significantly during the 52 weeks of the study.

The Treating to Target in Type 2 Diabetes (4-T) study revealed that the addition of analog biphasic, prandial, or basal insulin to maximal tolerated doses of metformin or an SU in patients with suboptimal glycemic control was associated with clinically relevant and sustainable reductions in HbA1c.^3^ Moreover, the biphasic and prandial regimens lowered HbA1c to the same extent and to a greater degree than the basal regimens. However, the target HbA1c levels were achieved in a minority of patients.^6^ Prandial insulin lowered HbA1c to the same extent as biphasic insulin, but with twice the number of episodes of hypoglycemia and more frequent weight gain.^6^ In the present study, no increase in body mass was measured. Twice-daily administration of detemir has been reported to achieve better glycemic control than once-daily administration.^8^ These findings are consistent with those in the continuing 4-T study, in which midday prandial insulin was added to the biphasic regimen, while basal insulin was added at bedtime to the prandial regimen, and prandial insulin was added at each meal to the basal regimen, if the HbA1c level was >6.5%. When following these protocols, patients who added a basal or prandial insulin-based regimen to oral therapy achieved better HbA1c control than patients who added a biphasic insulin-based regimen.^9^ The use of a single administration of basal insulin, such as glargine, was reported to be equally as effective as thrice-daily prandial insulin lispro for the control of hyperglycemia, when used in combination with an AHA, as shown in the APOLLO study.^10^ The risk of hypoglycemia and body mass increase were similar in the APOLLO and 4-T studies.^11^ The present study did not directly compare the efficacy of twice-daily injections with once daily glargine; therefore, it is impossible to draw conclusions regarding which is superior.

According to the protocol included in the ADA/EASD statement, patients with T2DM should be treated with basal insulin plus AHA (BOT) or multiple injections of rapid-acting insulin when the primary target of HbA1c <7% is not achieved after 3 months of treatment with metformin plus an SU, dipeptidyl peptidase-4 inhibitor, or pioglitazone. There were no differences between these options in the number of patients achieving the target, but more patients being treated with multiple injections of insulin experienced hyperglycemia.^4^

The present study showed significant differences in the effects of twice-daily injections of pre-mixed insulin, rapid-acting insulin plus an SU, and long-acting insulin plus a glinide on HbA1c. To prevent hyperglycemia in the morning, twice-daily injections of pre-mixed insulin are superior to twice-daily rapid-acting or long-acting insulin, without any increase in the frequency of hypoglycemia. After 12 weeks, glycemic rescue and lunch-time insulin were being administered, and the doses of insulin being administered had been increased in the Aspart and Detemir group, whereas no statistically significant increases in insulin dose had been necessary in the Mix group. Given that no significant increases in body mass occurred, the Mix group might have demonstrated superior effects on HbA1c compared with the Aspart and Detemir groups because an SU could not replace the effects of the long-acting insulin in the Aspart group, and the glinide could not replace the rapid-acting insulin effects in the Detemir group. The combinations of twice-daily injections of insulin with an SU or a glinide resulted in reductions in HbA1c, but these were below those required to achieve the target HbA1c in most of the participants. However, the number of participants was too small to detect statistically significant differences among the groups.

The principal limitation of the present study was the small number of participants. The low level of recruitment may be explained by the psychological barrier regarding the self-administration of insulin. However, the introduction of long-acting insulin into clinical practice makes it easier to persuade patients to start insulin in addition to AHAs; however, the increase in body mass and the modest additional effect on HbA1c limit its added value in the clinic. Further studies are required to confirm the superiority of multiple injections of insulin over BOT.

## Conclusions

Twice-daily injections of a premix are equally or more effective against hyperglycemia and similarly safe compared with twice-daily injections of aspart plus an SU and detemir plus a glinide in patients being treated with AHAs. Early and clinically meaningful reductions in HbA1c were achieved using twice-daily injections of premix, aspart plus an SU, or detemir plus a glinide, without severe hypoglycemia or an increase in body mass. However, we did not identify differences among the three insulin regimens, probably due to the small size of the study.

## Figures and Tables

**Table1:** Patient characteristics and disposition

Group	Total	Aspart	Mix	Detemir
Patients enrolled	31	13	9	9
Patients completing the trial	28	12	8	8
Reasons for lack of completion	3	1 lost to follow-up	1 discontinued insulin at week 15	1 lost to follow-up
Age (years)	57.5±10.4	57.5±6.5	63.4±12.0	51.8±11.1
Sex (male:female)	24:7	8:5	7:2	9:0
BMI (kg/m^2^)	23.1±4.76	23.9±4.73	22.6±5.14	22.5±4.82
Disease duration (years)	12.7±8.5	12.0±9.4	13.1±8.3	13.3±8.3
Treatment (αGI/BG/pioglitazone/DPP4i)	10/17/14/3	3/8/6/3	3/5/5/0	4/4/3/0
Retinopathy (none/simple/pre-proliferative/proliferative/undetermined)	19/6/2/1/3	9/1/0/1/2	6/1/1/0/1	4/4/1/0/0
Nephropathy stage (0/I/II/III/IV/V/undetermined)	15/3/10/1/0/0/2	7/1/5/0/0/0/0	4/1/3/0/0/0/1	4/1/2/1/0/0/1
Neuropathy (none/plus/undetermined)	17/9/5	7/4/2	5/2/2	5/3/1
Ischemic heart disease (none/OMI/Angina)	26/2/3	12/1/0	6/1/2	8/0/1
Stroke (none/infarction/stenosis/undetermined)	26/2/1/2	10/1/0/2	7/1/1/0	9/0/0/0
ASO (absent/present)	29/2	13/0	8/1	8/1
Gangrene (absent/present)	30/1	12/1	9/0	9/0

Data are mean±standard deviation (SD) or number. BMI: body mass index, αGI: α-glucosidase inhibitor, BG: biguanide, DPP4i: dipeptidyl-peptidase IV inhibitor, OMI: old myocardial infarction, ASO: arteriosclerosis obliterans. Mix: twice-daily dosing of biphasic insulin aspart-30

**Table2:** HbA1c values during the study (%)

HbA1c at each time point	0 weeks	12 weeks	24 weeks	52 weeks
Aspart	9.86±1.71 (13)	8.34±1.56* (12)	8.28±1.46* (12)	8.43±1.69* (12)
Mix	9.24±1.14 (9)	7.51±0.82*^†^ (9)	7.66±1.33* (9)	7.95±1.40* (8)
Detemir	11.26±1.81 (9)	9.30±1.56* (8)	9.03±1.86* (8)	8.33±1.10*^#^ (8)
Change in HbA1c		12 weeks *vs.* 0 weeks	24 weeks *vs.* 0 weeks	52 weeks *vs.* 0 weeks
Aspart		–1.52±1.69	–1.58±1.74	–1.43±2.07
Mix		–1.73±1.11	–1.59±1.07	–1.46±1.42
Detemir		–1.98±1.58	–2.21±2.30	–2.94±1.88

Data are mean±SD. * *p*<0.05 *vs.* 0 weeks, #*p*<0.05 *vs.* 12 weeks, ^†^ *p*<0.05 *vs.* Detemir at 12 weeks. Mix: twice-daily dosing of biphasic insulin aspart-30. The numbers in brackets are subjects.

**Table3:** Achievement of HbA1c values <7.4%

Time point	12 weeks	24 weeks	52 weeks
Aspart	16.7% (2/12)	16.7% (2/12)	16.7% (2/12)
Mix	55.6% (5/9)	33.3% (3/9)	37.5% (3/8)*
Detemir	25% (2/8)	12.5% (1/8)	12.5% (1/8)

Data are the percentage (number) of patients. * One patient in the Mix group stopped insulin after 24 weeks after achieving normal HbA1c. Mix: twice-daily dosing of biphasic insulin aspart-30.

**Table4:** Body weight at each time point

	0 weeks	12 weeks	24 weeks	52 weeks
Aspart	63.6±17.0 (13)	62.6±14.1 (11)	63.3±14.7 (11)	67.9±18.8 (11)
Mix	58.3±19.0 (9)	63.4±22.0 (7)	63.4±20.7 (7)	63.6±19.8 (7)
Detemir	63.2±18.7 (9)	62.9±20.6 (7)	63.7±18.4 (8)	57.3±16.0 (6)

Data are mean±SD, in kg. The numbers in brackets are subjects measured. Statistical analysis was performed by one-way ANOVA, followed by Wilcoxon/Kruskal-Wallis analysis. Mix: twice-daily dosing of biphasic insulin aspart-30.

**Table5:** Daily dose of insulin (U) at each time point

	0 weeks	12 weeks	24 weeks	52 weeks
Aspart	7.8±3.2	10.4±6.8	11.1±6.5*	13.4±10.6
Mix	15.2±7.4^†^	15.7±8.4	18.5±9.5	18.0±7.6
Detemir	12.9±5.2	16.3±4.8*	17.1±4.9	17.6±4.7*

Data are mean±SD. * *p*<0.05 *vs*. 0 weeks. ^†^ *p*<0.05 vs. Aspart at 0 weeks. Mix: twice-daily dosing of biphasic insulin aspart-30.

**Table6:** Lipid profile, blood chemistry, serum electrolytes, and hematologic indices at each time point

		0 weeks	12 weeks	24 weeks	52 weeks
LDL-cholesterol (mg/dl)	Aspart	104.0±31.1	97.8±43.2	105.2±48.0	92.8±22.3
	Mix	104.5±16.0	90.9±15.2	85.4±23.1	103.6±20.7
	Detemir	114.6±39.7	111.5±28.6	112.3±27.4	100.3±26.3*
HDL-cholesterol (mg/dl)	Aspart	64.7±16.0	64.3±8.7	64.3±10.9	63.0±7.9
	Mix	60.1±20.8	64.6±22.3	66.4±23.6	64.8±20.9
	Detemir	58.3±12.7	65.1±13.4	64.9±14.8	57.4±20.0
Triglyceride (mg/dl)	Aspart	123.1±93.7	153.2±111.0	162.8±145.5	151.6±125.8
	Mix	140.4±88.9	99.7±44.0	124.9±90.4	98.9±47.5
	Detemir	134.3±46.3	94.8±51.2*	110.3±52.6	104.5±56.6
Blood chemistry					
AST (U/ml)	Aspart	19.7±4.2	18.2±4.2	18.8±4.3	20.0±6.4
	Mix	21.8±8.7	21.0±5.1	27.6±13.2	23.3±7.5
	Detemir	15.3±4.2	16.3±3.6	17.9±4.8	18.1±4.4*
ALT (U/ml)	Aspart	23.2±9.7	19.6±7.9*	20.6±10.3	21.3±11.6
	Mix	24.1±11.7	18.6±7.2	30.1±25.1	23.3±8.9
	Detemir	19.9±6.7	15.4±4.3*	17.5±4.7	16.8±5.0
Albumin (mg/dl)	Aspart	4.33±0.51	4.42±0.47	4.45±0.41*	4.32±0.32
	Mix	4.06±0.53	4.22±0.45	4.10±0.66	4.07±0.64*
	Detemir	4.14±0.55	4.06±0.39^#^	4.29±0.42	4.01±0.39^#^
BUN (mg/dl)	Aspart	15.8±4.0	15.2±3.0	15.6±3.4	14.9±2.2
	Mix	16.0±7.1	14.3±3.1	15.4±4.2	16.8±7.2
	Detemir	14.4±7.5	15.6±4.2	14.6±3.3	14.6±4.4
Creatinine (mg/dl)	Aspart	0.63±0.18	0.64±0.18	0.65±0.20	0.64±0.20
	Mix	0.71±0.29	0.75±0.26	0.78±0.29	0.79±0.32
	Detemir	0.71±0.13	0.66±0.18	0.70±0.13	0.76±0.15
Serum electrolytes					
Na (mEq/l)	Aspart	138.6±1.9	139.7±3.0	139.4±2.4	139.4±2.2
	Mix	136.0±4.2	137.1±5.0	135.9±6.4	137.2±3.5
	Detemir	138.1±2.6	139.4±3.0	141.1±1.4*	140.0±1.9*
K (mEq/l)	Aspart	4.15±0.31	4.20±0.21	4.18±0.29	4.21±0.29
	Mix	4.07±0.74	4.09±0.76	4.41±0.67	4.30±0.40^#^
	Detemir	4.12±0.37	4.14±0.65	4.16±0.47	4.11±0.56
Cl (mEq/l)	Aspart	102.3±2.7	103.7±3.0	103.4±3.8	102.5±3.4
	Mix	100.0±4.1	98.5±4.4	99.3±5.2	99.6±5.4
	Detemir	102.1±2.7	103.7±2.1	103.5±1.9	102.5±1.8
Ca (mg/dl)	Aspart	9.6±0.4	9.5±0.3	9.6±0.4	9.6±0.3
	Mix	9.8±0.5	9.7±0.2	9.5±0.4	9.9±0.4
	Detemir	9.1±0.2	9.6±0.3*	9.8±0.2*	9.5±0.4
Complete blood count					
RBC (×10^4^)	Aspart	478±32	467±46	473±34	470±50
	Mix	432±50	448±53	447±64	427±81
	Detemir	457±26	456±20	472±26*	454±30
Hb (g/dl)	Aspart	14.6±1.3	14.3±1.5	14.4±1.5	14.4±1.9
	Mix	13.4±1.0	14.0±1.3	13.8±1.4	12.9±1.5
	Detemir	14.2±0.9	13.8±0.8	14.4±0.9	14.0±1.2
Ht (%)	Aspart	43.5±3.0	42.9±3.9	43.5±3.9	42.9±4.6
	Mix	40.0±2.8	41.2±3.8	40.5±4.2	38.7±4.7
	Detemir	42.8±3.8	41.7±3.2	43.3±2.6	41.84±4.2
WBC	Aspart	6,555±1,981	6,517±1,522	6,308±1,436	6,375±1,640
	Mix	6,150±1,818	7,357±4,178	6,675±2,186	5,971±1,614
	Detemir	6,233±1,336	6,088±1,575	6,813±1,760*	6,525±1,483
Platelets (×10^4^)	Aspart	20.4±5.0	21.7±5.5	21.2±4.4	21.9±4.7*
	Mix	20.3±2.9	21.9±6.3	20.3±3.1	20.3±4.1
	Detemir	20.2±4.7	21.2±4.7	20.8±6.4	20.5±4.1

Data are mean±SD. * *p*<0.05 *vs.* 0 w, ^#^ *p*<0.05 *vs.* 24 w. w: week, SD: standard deviation, LDL: low-density lipoprotein, HDL: high-density lipoprotein, AST: aspartate aminotransferase, ALT: alanine aminotransferase, BUN: blood urea nitrogen, RBC: red blood cell, Hb: hemoglobin, Ht: hematocrit, WBC: white blood cell. Mix: twice-daily dosing of biphasic insulin aspart-30.
